# Prognostic value of left atrial volume index in patients with rheumatic mitral stenosis

**DOI:** 10.1002/clc.23544

**Published:** 2021-01-06

**Authors:** In‐Jeong Cho, Hyeonju Jeong, Hyuk‐Jae Chang

**Affiliations:** ^1^ Division of Cardiology, Department of Internal Medicine Ewha Womans University Seoul Hospital, College of Medicine, Ewha Womans University Seoul South Korea; ^2^ Division of Cardiology, Department of Internal Medicine Myungji Hospital Goyang South Korea; ^3^ Division of Cardiology, Severance Cardiovascular Hospital Yonsei University College of Medicine Seoul South Korea

**Keywords:** left atrium, mitral stenosis, prognosis

## Abstract

**Background:**

The significance of left atrial volume index (LAVI) for predicting outcomes in patients with mitral stenosis (MS) has been unclear, even though rheumatic MS is known to be associated with left atrium enlargement and functional deterioration.

**Hypothesis:**

The current study aimed to investigate the prognostic value of LAVI, based on the severity in patients with rheumatic MS.

**Methods:**

We retrospectively reviewed 611 patients with pure rheumatic MS. The prognostic value of LAVI and the effect of MS severity on the prognostic value of LAVI for events were evaluated. The events were defined as a composite end‐point that included all‐cause death, heart failure admission, mitral valve replacement, percutaneous mitral valvuloplasty, and stroke.

**Results:**

There were 236 (38.6%) overall events during a median follow‐up of 8 months. The optimal LAVI cutoff for the prognostic threshold was 57 ml/m^2^. The MS severity had a significant effect on the prognostic value of LAVI. A LAVI >57 ml/m^2^ was a prognostic value for events in progressive MS (hazard ratio [HR]: 2.40, 95% confidence interval [CI]: 1.41–5.40, *p* = .004) and in patients with severe MS (HR: 1.70, 95% CI: 1.06–2.74, *p* = .029), but it was not prognostic in patients with very severe MS (HR: 1.02, 95% CI: 0.56–1.84, *p* = .955).

**Conclusions:**

The prognostic value of LAVI varies and is dependent on the MS severity. A LAVI >57 mL/m^2^ was independently associated with poor outcomes in patients with progressive MS, while this association was minimized in patients with severe MS.

## 1 INTRODUCTION

The left atrial volume index (LAVI) is a known prognostic marker for cardiovascular outcomes in various cardiovascular diseases, including heart failure, hypertrophic cardiomyopathy, and ischemic heart disease, as well as in the general population.^1^, ^2^, ^3^, ^4^ The prognostic role of LAVI in valvular heart disease has also been applied in patients with mitral regurgitation (MR),^5^, ^6^ aortic stenosis,^7^, ^8^, ^9^ and aortic regurgitation.^10^ However, the value of LAVI for predicting outcomes in patients with mitral stenosis (MS) has been unclear, even though rheumatic MS is known to be closely associated with left atrium enlargement, stiffening, and functional deterioration.^11^ One report has indicated that LAVI did not predict clinical outcomes in patients with MS.^12^

We recently found that LAVI can act as a prognostic marker for outcomes in patients with progressive MS, which is defined as MS patients with the mitral valve area (MVA) larger than 1.5 cm^2^.^13^ Progressive MS is a less severe stage during disease progression in MS. Therefore, we hypothesized that the prognostic value of LAVI for MS would differ according to the severity of the disease, and this might cause discordant results for the prognostic value of LAVI in patients with MS. We aimed to investigate the effect of MS severity on the prognostic value of LAVI in a large cohort of patients with MS, categorized as progressive MS, severe MS, and very severe MS. We additionally sought to assess associated factors for an enlarged left atrium other than the severity of MS in those patients.

## 2 METHODS

### 2.1 Study population

We analyzed patients with rheumatic MS who underwent echocardiography between 2006 and 2015 at a tertiary referral center for valvular heart disease in Korea. Exclusion criteria were as follows: patients with >1+ MR, >1+ aortic regurgitation and/or more than mild aortic stenosis, patients with congenital or myopathic lesions that could affect pulmonary artery pressure, patients with a history of prior percutaneous mitral valvuloplasty (PMV), those who had undergone planned mitral valve replacement (MVR) or PMV before the echocardiographic examination, and those who received MVR or PMV within 30 days after the index echocardiography examination. Demographic characteristics including age, sex, anticoagulation, history of prior stroke, and body surface area were confirmed by chart review. Systolic and diastolic blood pressure were measured before the echocardiography. Therefore, in total, 611 patients with pure rheumatic MS were included. This study was approved by the ethical committee of Yonsei University, Severance Hospital, Seoul, Korea. The need to obtain informed consent was waived for the retrospectively obtained data for this non‐interventional study.

### 2.2 Echocardiography

Two‐dimensional and Doppler echocardiography were performed according to the American Society of Echocardiography (ASE) guidelines.^14^ Left ventricular (LV) end‐diastolic dimension (EDD), LV end‐systolic dimension (ESD), septal wall thickness, and posterior wall thickness were measured from the M‐mode. LV ejection fraction (EF) was calculated using LV EDD and ESD. LV mass was calculated using the formula, according to the ASE guidelines. The LV mass index was defined as an LV mass indexed for body surface area. LV hypertrophy was defined as LV mass index ≥115 g/m^2^ for men and ≥95 g/m^2^ for women.^14^ The left atrial volume was calculated using the biplane area‐length method according to ASE guidelines.^14^ Two‐dimensional volumetric measurements were based on left atrial areas measures by tracings of the blood‐tissue interface and left atrial lengths on apical four‐ and two‐chamber views (Figure S1). LAVI was defined as left atrial volume indexed for body surface area. The MVA was assessed by two‐dimensional planimetry. MS was categorized according to the MVA; progressive MS (1.5 cm^2^ < MVA ≤2.0 cm^2^), severe MS (1.0 cm^2^ < MVA ≤1.5 cm^2^), and very severe MS (MVA ≤1.0 cm^2^).^15^

The mean diastolic transmitral pressure gradient was measured from a continuous wave Doppler signal across the mitral valve by tracing its envelope. The calculated systolic pulmonary artery pressure was defined as 4 × (maximum velocity of the tricuspid regurgitant jet)^2^ + right atrial pressure. Right atrial pressure was estimated by measuring the inferior vena cava diameter.^16^ Stroke volume was calculated using the LV outflow tract diameter and the LV outflow tract flow pulsed‐wave Doppler signal. The stroke volume index was defined as stroke volume indexed for body surface area.

### 2.3 Study endpoint

Patients were followed across a median of 41 months (Interquartile range: 8–84 months) for a composite end‐point that included all‐cause death, inpatient admissions for heart failure, MVR, PMV, and incidence of stroke. The occurrence of any of the clinical events was ascertained by a review of hospital records and by telephone interviews, as necessary.

### 2.4 Statistics

Demographic characteristics are reported as percentages or as the mean ± SD. The patient groups were compared using chi‐square statistics for categorical variables and the Student's *t*‐test for continuous variables. Receiver operating characteristic (ROC) curves were used to determine the sensitivity and specificity of LAVI in predicting the primary outcomes and to determine the optimal cut‐off value for continuous variables. Univariable and multivariable Cox proportional‐hazards regression models reporting the hazard ratio (HR) and 95% confidence interval (CI) was employed to determine potential useful variables for predicting event‐free survival following echocardiography. Variables with statistical significance in univariable analysis were entered into the multivariable Cox proportional hazard model, as well as age and sex. Kaplan–Meier survival curves were employed to plot all events according to the time‐to‐first event. To determine potential independent associations between variables and LAVI, binary logistic regression was applied. Variables displaying statistical significance in univariable analysis were entered into a multivariable binary logistic regression model, reporting the odds ratio (OR) and 95% CI. A *p* value <.05 was considered statistically significant.

## 3 RESULTS

Table 1 demonstrates the baseline characteristics of the study population. There were 207 patients with progressive MS, 281 patients with severe MS, and 123 patients with very severe MS from the overall 611 patients with MS. The mean age was 60 ± 12, and 76.4% of the patients were women. Patients with very severe MS were younger than those with progressive and severe MS (56 ± 12 years vs. 62 ± 12 years vs. 60 ± 12 years). There were no significant differences in the sex between the groups. LV hypertrophy was more common in patients with progressive and severe MS compared to those with very severe MS (37.7% vs. 32.7% vs. 16.2%). The outcomes of the population are shown in Table S1. There were 236 (38.6%) overall events, including 3 cardiac deaths (0.5%), 24 heart failure admissions (3.9%), 129 MVRs (21.1%), 51 PMVs (8.3%), and 29 strokes (4.7%). There were 33 (15.9%) events in patients with progressive MS, 118 (42.0%) events in patients with severe MS, and 85 (69.1%) events in patients with very severe MS.

**TABLE 1:** Baseline characteristics

Variable	Overall (*n* = 611)	MS grade		
		Progressive (*n* = 207)	Severe (*n* = 281)	Very severe (*n* = 123)
Demographics				
Age, years	60 ± 12	62 ± 12	60 ± 12	56 ± 12^a^ ^,^ ^b^
Female sex, *n* (%)	467 (76.4)	156 (75.4)	217 (77.2)	94 (76.4)
AF, *n* (%)	327 (53.5)	86 (41.5)	160 (56.9)^a^	81 (65.8)^a^
Anticoagulation, *n* (%)	339 (55.5)	75 (36.2)	174 (61.9)	90 (73.1)^a^ ^,^ ^b^
Prior stroke, *n* (%)	88 (14.4)	21 (10.1)	43 (15.3)	24 (19.5)
Symptomatic, *n* (%)	209 (34.2)	55 (26.6)	100 (35.6)^a^	54 (43.9)^a^
Body surface area, m^2^	1.60 ± 0.16	1.63 ± 0.17	1.60 ± 0.16^a^	1.56 ± 0.14^a^ ^,^ ^b^
Systolic BP, mm Hg	119.5 ± 19.1	122.3 ± 22.1	118.0 ± 16.4	116.5 ± 16.7
Diastolic BP, mm Hg	74.7 ± 13.1	77.6 ± 15.0	72.6 ± 11.1^a^	72.8 ± 11.6^a^
Echocardiography				
LV EDD, mm	48.1 ± 4.5	48.6 ± 4.7	48.2 ± 4.4	46.9 ± 4.3^a^ ^,^ ^b^
LV ESD, mm	32.3 ± 4.2	32.5 ± 4.5	32.4 ± 4.1	31.9 ± 3.8
LV EF, %	64.0 ± 7.2	64.8 ± 7.6	63.8 ± 7.3	63.3 ± 6.2
LV mass index, g/m^2^	91.1 ± 23.0	95.8 ± 22.3	91.3 ± 21.7^a^	82.8 ± 24.8^a^ ^,^ ^b^
LAVI, ml/m^2^	68.0 ± 32.6	54.8 ± 24.4	72.5 ± 30.8^a^	83.7 ± 39.3^a^ ^,^ ^b^
MVA, cm^2^	1.33 ± 0.36	1.74 ± 0.14	1.24 ± 0.14^a^	0.82 ± 0.14^a^ ^,^ ^b^
MDPG, mm Hg	6.0 ± 3.2	3.6 ± 1.1	5.9 ± 2.2^a^	10.1 ± 3.4^a^ ^,^ ^b^
SPAP, mm Hg	34.5 ± 11.3	30.3 ± 8.6	33.8 ± 9.3^a^	43.6 ± 13.9^a^ ^,^ ^b^
Stroke volume index, ml/m^2^	39.0 ± 8.7	39.9 ± 9.1	38.8 ± 8.7	37.9 ± 8.0
LV hypertrophy, *n* (%)	190 (31.1)	78 (37.7)	92 (32.7)	20 (16.2)^a^ ^,^ ^b^

^a^ *p* value <.05 compared to progressive MS.

^b^ *p* value <.05 compared to severe MS.

Abbreviations: AF, atrial fibrillation; BP, blood pressure; EDD, end‐diastolic dimension; EF, ejection fraction; ESD, end‐systolic dimension; LAVI, left atrial volume index; LV, left ventricular; MDPG, mean diastolic pressure gradient; MS, mitral stenosis; MVA, mitral valve area; SPAP, systolic pulmonary artery pressure.

The optimal cut‐off value for predicting overall events according to the ROC curves for the LAVI was 57 ml/m^2^, in which the area under curve (AUC) was 0.657, the sensitivity was 76.2% and specificity was 50.2%. We categorized patients into two LAVI groups as follows: LAVI >57 ml/m^2^ and LAVI ≤57 ml/m^2^ for a comparison. To confirm optimal LAVI cut‐offs at each MS severity grade, there was additional investigation of the optimal cut‐off value for predicting overall events according to the ROC curves for LAVI in each of the progressive, severe, and very severe MS groups. The optimal cut‐off for LAVI was 51 ml/m^2^ for progressive MS (AUC: 0.693, sensitivity = 77.1%, specificity = 57.9%), 52 ml/m^2^ for severe MS (AUC: 0.531, sensitivity = 81.2%, specificity = 29.6%), and 58 ml/m^2^ for very severe MS (AUC: 0.529; sensitivity = 73.6%, specificity = 26.4%).

**FIGURE 1:**
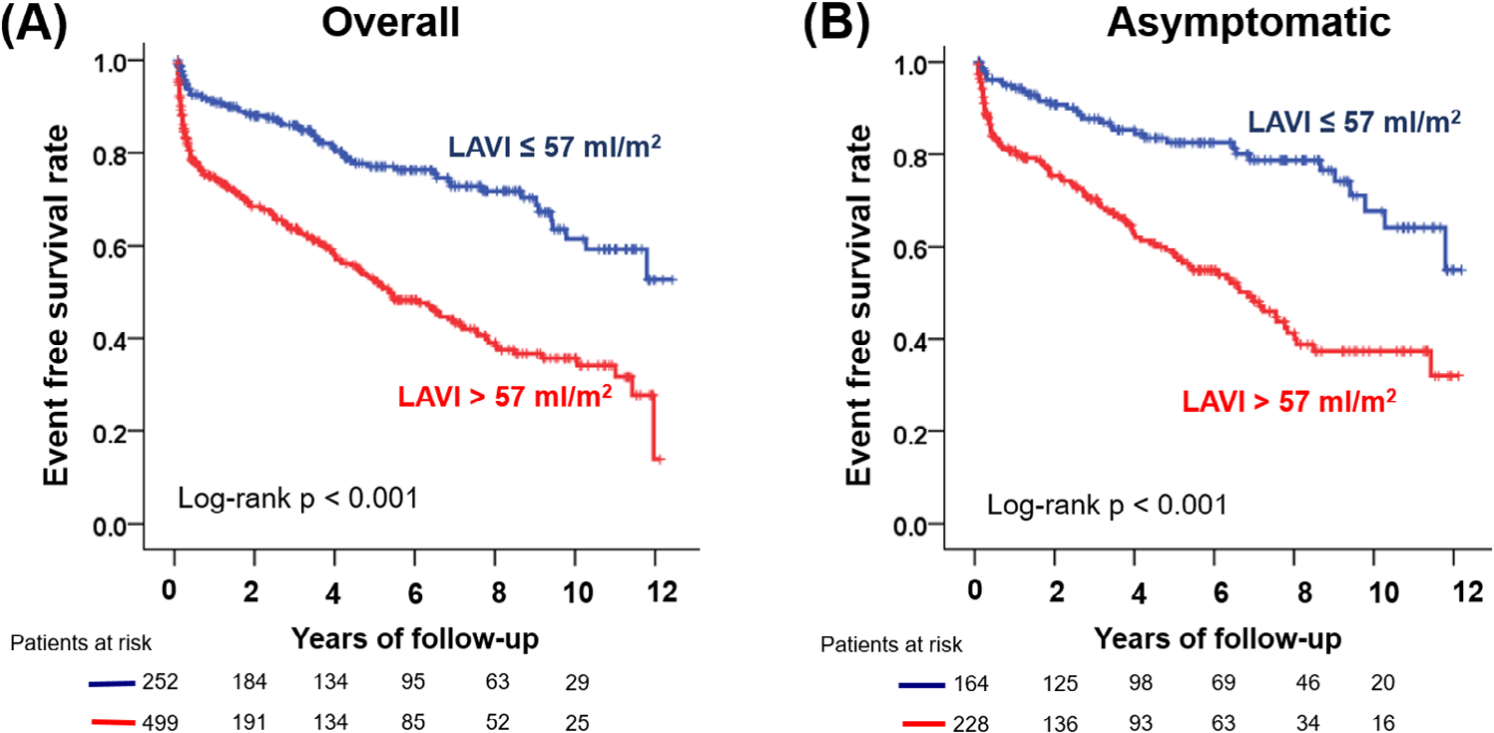
Kaplan–Meier curve to demonstrate the outcomes according to the left atrial volume index. (A) overall population and (B) asymptomatic patients

Table 2 demonstrates determinants of clinical outcomes. In univariable analysis, atrial fibrillation (HR: 1.46, 95% CI: 1.12–1.90, *p* = .005), presence of symptom (HR: 1.41, 95% CI: 1.09–1.83, *p* = .009), LAVI >57 ml/m^2^ (HR: 2.01, 95% CI: 1.51–2.70, *p* < .001), and MS grade (HR: 2.63, 95% CI: 1.79–3.88, *p* < .001 for severe MS; HR: 4.34, 95% CI: 2.90–6.48, *p* < .001 for very severe MS) were associated with the incident of events. However, a LAVI >57 ml/m^2^ (HR: 1.41, 95% CI: 1.01–1.98, *p* = .048) and MS grade (HR: 2.29, 95% CI: 1.54–3.41, *p* < .001 for severe MS; HR: 3.44, 95% CI:2.23–5.32, *p* < .001 for very severe MS) were independently associated with events, after adjusting for other confounding factors, suggesting that LAVI was the only predictor for events, excluding MS severity. Figure 1 demonstrates the Kaplan–Meier curve for outcomes, according to LAVI, in overall and asymptomatic patients. A LAVI >57 ml/m^2^ was associated with poor outcomes in the overall population and in a subgroup of asymptomatic patients (all log‐rank *p* < .001).

**TABLE 2:** Determinants for overall events

Variable	Univariable			Multivariable		
	HR	95% CI	*p* value	HR	95% CI	*p* value
Age	1.00	0.99–1.01	.387	1.00	0.99–1.01	.780
Sex	0.99	0.73–1.34	.954	0.98	0.72–1.32	.882
AF	1.46	1.12–1.90	.005	1.12	0.82–1.52	.483
Symptomatic	1.41	1.09–1.83	.009	1.26	0.97–1.63	.088
LAVI >57 ml/m^2^	2.01	1.51–2.70	<.001	1.41	1.01–1.976	.048
LVEF >50%	0.65	0.36–1.20	.169	‐		
MS grade						
Progressive	1.00	‐	‐	1.00	‐	‐
Severe	2.63	1.79–3.88	<.001	2.29	1.54–3.41	<.001
Very severe	4.34	2.90–6.48	<.001	3.44	2.23–5.32	<.001

Abbreviations: AF, atrial fibrillation; CI, confidence interval; HR, hazard ratio; LAVI, left atrial volume index; LVEF, left ventricular ejection fraction; MS, mitral stenosis.

**FIGURE 2:**
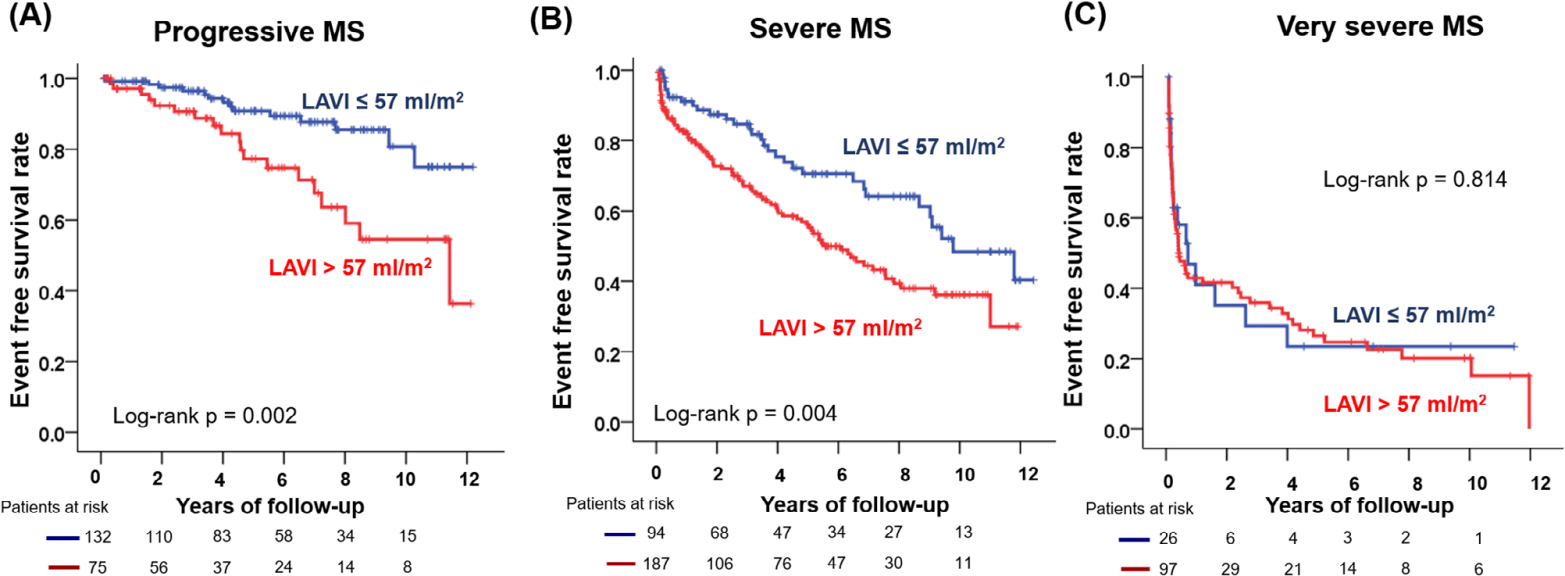
Kaplan–Meier curve to demonstrate the outcomes according to the left atrial volume index. (A) progressive MS, (B) severe MS, and (C) very severe MS. MS, mitral stenosis

Because interaction testing confirmed the effect of MS severity on the LAVI prognostic value (interaction *p* < .001), we evaluated the LAVI prognostic value according to the MS severity (Table 3). In patients with progressive MS, a LAVI >57 ml/m^2^ was a prognostic value for events (HR: 2.40, 95% CI: 1.41–5.40, *p* = .004), even after adjusting for age, sex, atrial fibrillation, symptom, and MVA. In patients with severe MS, this association was reduced but still existed (HR: 1.70, 95% CI: 1.06–2.74, *p* = .029), while a LAVI >57 ml/m^2^ was not prognostic for events in patients with very severe MS (HR: 1.02, 95% CI: 0.56–1.84, *p* = .955). Figure 2 demonstrates the Kaplan–Meier curve for outcomes according to LAVI in each group: progressive MS, severe MS, and very severe MS, respectively. A LAVI >57 ml/m^2^ was associated with poor outcomes in patients with progressive MS (log‐rank *p* = .002) and in patients with severe MS (log‐rank *p* = .004), whereas this association was not found in patients with very severe MS (log‐rank *p* = .814).

**TABLE 3:** Prognostic value^a^ of left atrial volume index by mitral stenosis severity

	HR for events with LAVI >57 ml/m^2^	95% CI	*p* value
Progressive	2.40	1.41–5.40	.004
Severe	1.70	1.06–2.74	.029
Very severe	1.02	0.56–1.84	.955

^a^ Cox proportional hazards model for time to the event, adjusted for age, sex, atrial fibrillation, symptom, and mitral valve area.

Abbreviations: CI, confidence interval; HR, hazard ratio; LAVI, left atrial volume index.

Echocardiographic factors that correlated with an LAVI >57. ml/m^2^ in patients with MS are shown in Table S2. In univariable analysis, LV EDD and ESD, LV EF, LV hypertrophy, and MS grade were all associated with a LAVI >57 ml/m^2^. However, LV hypertrophy (OR: 2.09, 95% CI: 1.35–3.22, *p* = .001) and MS grade (OR: 4.12, 95% CI: 2.75–6.18, *p* < .001 for severe MS; OR: 8.93, 95% CI: 5.11–15.62, *p* < .001 for very severe MS) were independently associated factors for LAVI >57 ml/m^2^, even after adjusting for various echocardiographic variables. To assess association of LAVI and LV hypertrophy at each MS severity category, the echocardiographic factors that correlated with a LAVI >57 ml/m^2^ were assessed in each MS severity category, respectively. LV hypertrophy was independently associated with LAVI >57 ml/m^2^ in progressive MS (OR: 2.22, 95% CI: 1.26–3.90, *p* = .006) and severe MS (OR: 1.43, 95% CI: 1.00–2.06, *p* = .048), even with adjustments for LV EDD, LV ESD, and LV EF; however, it did not show statistically significant association in very severe MS (OR: 2.78, 95% CI: 0.60–12.91, *p* = .189).

## 4 DISCUSSION

The main findings of this study are that (1) the prognostic value of LAVI varies and is dependent upon the MS severity and (2) a large LAVI was independently associated with poor outcomes in patients with progressive MS, while this association weakens in patients with severe MS and the prognostic value of LAVI was not seen in patients with very severe MS.

### 4.1 Left atrium in mitral valve disease

LAVI is a known prognostic marker for cardiovascular outcomes in various cardiovascular diseases that involve the left ventricle, as well as in the general population.^1^, ^2^, ^3^, ^4^ This is primarily because left atrial enlargement is a marker for LV diastolic dysfunction and elevated filling pressure. An increase in LV filling pressure and left atrial pressure results in enlargement of left atrial size and functional deterioration.

Mitral valve disease, either stenosis or regurgitation, is the main cause for left atrial enlargement through elevation of left atrial pressure. Therefore, left atrial enlargement usually reflects the severity and duration of mitral valve disease. However, the predictive role of LAVI in MS patients has been less clear,^11^ because it differs from the well‐known prognostic value of LAVI in patients with MR,^5^, ^6^ although a giant left atrium has been frequently noted in patients with long‐standing rheumatic MS.^17^

Some reports have indicated that MR rather than MS was an important predictor of left atrial reverse remodeling after mitral valve surgery,^18^ suggesting differences in left atrial remodeling and the mechanics of MS compared to MR. The plausible explanation for the difference in left atrial remodeling between MS and MR is that increased left atrial work caused by pressure overload in MS results in severe left atrial fatigue and failure over time.^19^ Electron microscopy study has also reported that endothelial cell surface changes are more severe in patients with MS than in those with MR, which suggests severe endothelial damage of the left atrial wall resulting from heavy pressure overload in patients with MS.^20^

Our results indicated that the prognostic role of LAVI was not found in patients with very severe MS of MVA ≤1.0 cm^2^, which is consistent with the study that showed a lack of prognostic value for LAVI in MS.^12^ However, our data also suggest that there is an interaction of MS severity in the prognostic role of LAVI. That is, as the MVA becomes smaller, the LAVI prognostic value becomes weaker. Interestingly, patients with progressive MS and severe MS, who had prognostic values for LAVI, were older than those with very severe MS. This fact suggests that those patients might be a subgroup of the population that have experienced less severe pressure overload in the left atrium over longer duration. Moreover, aging is a well‐known risk factor for LV diastolic dysfunction, and therefore, we can hypothesize that LV diastolic dysfunction might be a cause for left atrial enlargement in those subgroups of the population.

### 4.2 Left ventricle influence on the left atrium in MS

Despite the long‐held belief that rheumatic MS is an isolated disease of the stenotic mitral valve, several studies have suggested LV myocardial abnormalities in a subset of patients with rheumatic MS.^21^, ^22^, ^23^ We previously reported that LV diastolic dysfunction and increased LV diastolic pressure is a mechanism for the low‐gradient phenomenon in subsets of MS, which is commonly found in elderly patients with atrial fibrillation.^22^ From the current study, we also found that LV hypertrophy was independently associated with a large LAVI. Because LV hypertrophy is a well‐known cause of LV diastolic dysfunction and an increase in LV stiffness, we can speculate that LV diastolic dysfunction in elderly patients affects the left atrial pressure and volume even in MS patients, similar to other cardiovascular diseases involving the left ventricle.

Interestingly, the presence of LV hypertrophy was more prevalent in the order of progressive MS, severe MS, and very severe MS. Therefore, the influence of LV properties might be more prominent in progressive MS, which showed the most powerful prognostic value of the LAVI on outcomes in the MS subgroups. LV hypertrophy and associated LV diastolic dysfunction might aggravate left atrial enlargement, and accelerate poor clinical outcomes in MS patients with relatively larger MVAs. Therefore, LV hypertrophy and diastolic dysfunction can be a treatment target for patients with MS with MVA >1.0 cm^2^, especially in the elderly population with LV hypertrophy.^24^

Figure S2 demonstrates our hypothesis and a summary of determinants for LAVI and the prognostic value in patients with MS, according to the disease severity. To summarize, LAVI is determined by LV myocardial properties and MS severity, and the prognostic value decreases as the MVA decreases. For left atrial enlargement, LV myocardial property is important in progressive MS, whereas its importance weakens and MVA becomes more important in very severe MS.

### 4.3 Limitations

The main limitation of the current study is that the results of this study were based on retrospective analysis. However, patient medical records and echocardiography were carefully reviewed. We defined the presence of symptoms from clinical records, even though all medical records and echocardiography were reviewed carefully to minimize any bias. The choice to perform surgery or another intervention was made by each attending physician; therefore, it was not standardized and may have been influenced by the patient's LAVI. However, since LAVI is not included in the echocardiographic parameters for determining MS intervention in the current guideline,^15^, ^25^ the LAVI would not have had a significant effect on each physician's decision.

## 5 CONCLUSION

The prognostic value of LAVI varies and is dependent upon the MS severity. A LAVI >57 ml/m^2^ was independently associated with poor outcomes in patients with progressive MS, while this association weakened in patients with severe MS. The prognostic value of LAVI was not identified in patients with very severe MS. LAVI was independently associated with the presence of LV hypertrophy, suggesting the influence of LV myocardial properties on LAVI in patients with MS.

## CONFLICT OF INTEREST

The author declares that there is no conflict of interest that could be perceived as prejudicing the impartiality of the research reported.

## Supporting information


**Figure S1.** Two‐dimensional echocardiographic measurements for left atrial volume measurement.

**Figure S2.** Left atrial volume index determinants and the associated prognostic value in patients with mitral stenosis according to the disease severity.
The left atrial volume index is determined by left ventricular property and mitral stenosis severity, and the prognostic value decreased as the mitral valve area decreased.
LAVI, left atrial volume index; LV, left ventricle; MS, mitral stenosis.

**Table S1.** Population outcomes.
**Table S2.** Echocardiographic correlations with left atrial volume index >57 ml/m^2^ in patients with mitral stenosis.

## Data Availability

The data that support the findings of this study are available from the corresponding author upon reasonable request.
