# Supplementary figures and images for: Prognostic value of left atrial volume index in patients with rheumatic mitral stenosis
Source: Clin Cardiol. 2021 Jan 6;44(3):364–70. doi: 10.1002/clc.23544 (PMC7943912; doi:10.1002/clc.23544)

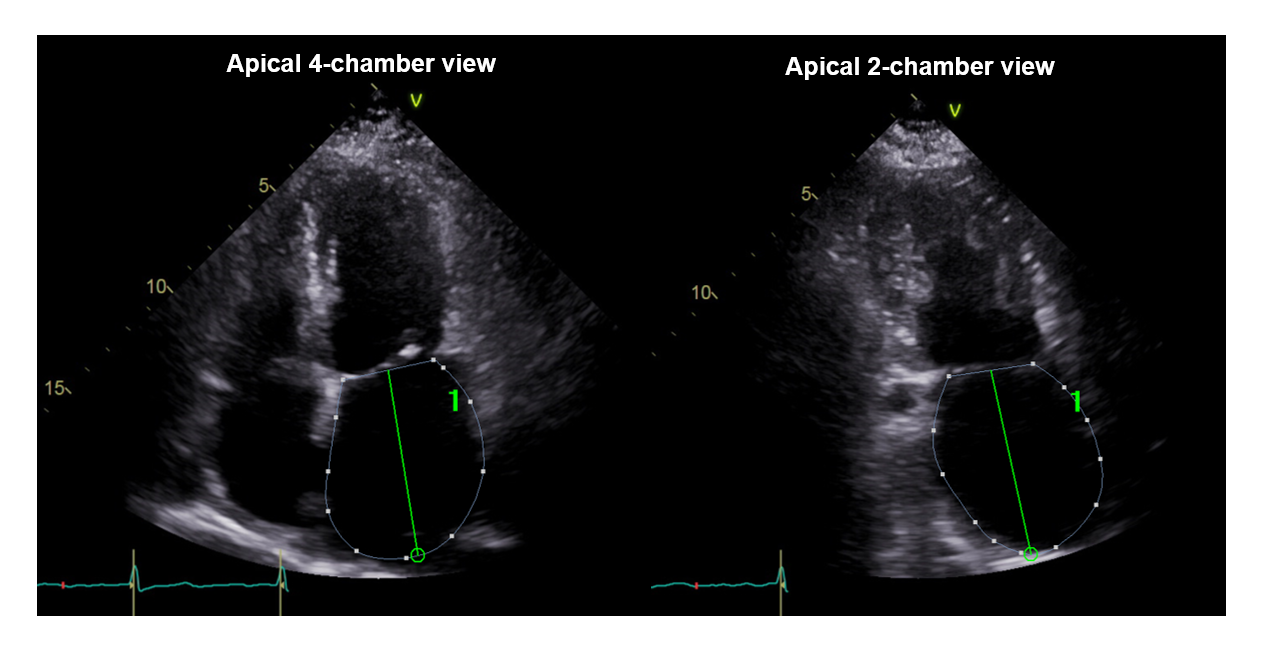

Supplement: Supplementary file 1 — Figure S1. Two‐dimensional echocardiographic measurements for left atrial volume measurement. [file CLC-44-364-s002.tif]

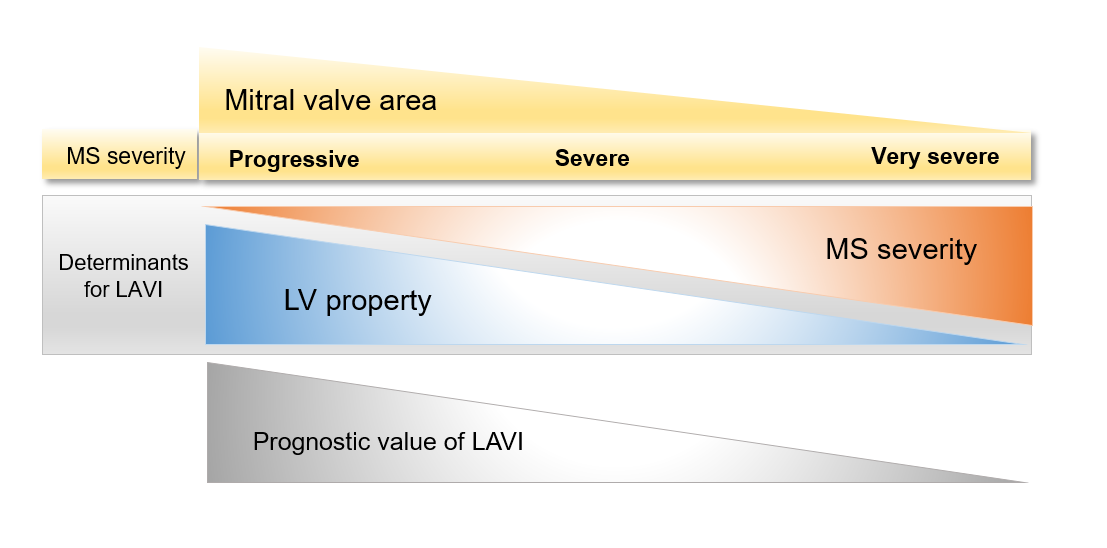

Supplement: Supplementary file 2 — Figure S2. Left atrial volume index determinants and the associated prognostic value in patients with mitral stenosis according to the disease severity. The left atrial volume index is determined by left ventricular property and mitral stenosis severity, and the prognostic value decreased as the mitral valve area decreased. LAVI, left atrial volume index; LV, left ventricle; MS, mitral stenosis. [file CLC-44-364-s003.tif]
