# Supplementary material for: Prognostic value of left atrial volume index in patients with rheumatic mitral stenosis
Source: Clin Cardiol. 2021 Jan 6;44(3):364–70. doi: 10.1002/clc.23544 (PMC7943912; doi:10.1002/clc.23544)
Supplement: Supplementary file 3 — Table S1. Population outcomes. Table S2. Echocardiographic correlations with left atrial volume index >57 ml/m2 in patients with mitral stenosis. [file CLC-44-364-s001.docx]

**Supplemental Table1.** Population outcomes.

**Supplemental Table2.** Echocardiographic correlations with left atrial volume index > 57 mL/m^2^ in patients with mitral stenosis.

**Supplemental Table1.** Population outcomes.

| Event | Overall  (n = 611) | MS grade | | |
| --- | --- | --- | --- | --- |
|  |  | Progressive  (n = 207) | Severe  (n = 281) | Very severe  (n = 123) |
| Overall, n (%) | 236 (38.6) | 33 (15.9) | 118 (42.0) | 85 (69.1) |
| Cardiac death, n(%) | 3 (0.5) | 2 (1.0) | 1 (0.4) | 0 (0) |
| HF admission, n (%) | 24 (3.9) | 9 (4.3) | 13 (4.6) | 2 (1.6) |
| MVR, n (%) | 129 (21.1) | 12 (5.8) | 62 (22.1) | 55 (44.7) |
| PMV, n (%) | 51 (8.3) | 1 (0.5) | 23 (8.2) | 27 (22.0) |
| Stroke, n (%) | 29 (4.7) | 9 (1.5) | 19 (1.3) | 1 (0.3) |

MS, mitral stenosis; HF, heart failure; MVR, mitral valve replacement; PMV, percutaneous mitral valvuloplasty.

**Supplemental Table2.** Echocardiographic correlations with left atrial volume index > 57 mL/m^2^ in patients with mitral stenosis.

| Variable | Univariable | | | Multivariable | | |
| --- | --- | --- | --- | --- | --- | --- |
|  | OR | 95% CI | P value | OR | 95% CI | P value |
| LV EDD, mm | 1.07 | 1.03-1.11 | < 0.001 | 0.88 | 0.72-1.06 | 0.184 |
| LV ESD, mm | 1.12 | 1.07-1.17 | < 0.001 | 1.35 | 1.02-1.79 | 0.039 |
| LV EF, % | 0.95 | 0.93-0.97 | < 0.001 | 1.05 | 0.96-1.16 | 0.288 |
| LV hypertrophy | 1.69 | 1.18-2.42 | 0.004 | 2.09 | 1.35-3.22 | 0.001 |
| MS grade |  |  |  |  |  |  |
| Progressive | 1.00 | - | - | 1.00 | - | - |
| Severe | 3.50 | 2.40-5.10 | < 0.001 | 4.12 | 2.747-6.18 | < 0.001 |
| Very severe | 6.57 | 3.91-11.02 | < 0.001 | 8.93 | 5.11-15.62 | < 0.001 |

OR, odds ratio; CI, confidence interval; LV, left ventricular; EDD, end-diastolic dimension; ESD, end-systolic dimension; EF, ejection fraction; MS, Mitral stenosis.
